# Post-Operative Sensitivity and Color Change Due to In-Office Bleaching With the Prior Use of Different Desensitizing Agents: A Systematic Review

**DOI:** 10.7759/cureus.24028

**Published:** 2022-04-11

**Authors:** Karishma Krishnakumar, Anita Tandale, Vini Mehta, Shruti Khade, Twinkle Talreja, Gaurav Aidasani, Anukriti Arya

**Affiliations:** 1 Department of Conservative Dentistry and Endodontics, Dr. D.Y. Patil Dental College & Hospital, Dr. D.Y. Patil Vidyapeeth, Pune, IND; 2 Public Health Dentistry, People's College Of Dental Science and Research Center (PCDS) People's University Campus, Bhopal, IND; 3 Department of Restorative Dentistry, School of Dentistry, University of California, California, USA; 4 Medical School, College of Health and Human Sciences, Swansea University, Wales, GBR

**Keywords:** colour alteration, in office bleach, vas, dentin desensitizing agents, tooth bleaching agents

## Abstract

Bleaching agents can cause certain surface alterations on the enamel, such as depressions, surface porosity, and surface irregularities; this makes the dentin more susceptible to post-operative tooth sensitivity (PoS). In addition, the presence of flawed or leaky restorations, gingival recession, or defects in the cementoenamel junction can also cause severe tooth sensitivity post tooth bleaching.Hence, the current study aimed to perform a systematic review to determine the efficacy of various desensitizing agents (DA) in managing post-operative tooth sensitivity and color alteration when applied before in-office bleaching procedures. Randomized clinical trials were searched to conduct an SR where the post-operative tooth sensitivity was evaluated after in-office bleaching with various desensitizing agents used before the procedure. Post-operative pain assessment was measured using the Visual Analogue Scale (VAS). Outcomes were evaluated up to an hour and 24 hours post the procedure. Out of 163 articles, only 13 titles were selected that met the eligibility criteria. Eight hundred and forty-one adult patients with vital pulp status were included. The participants receiving prior desensitizing agent applications reported significantly lower pain scores in the VAS reports. The most significant reduction of post-operative sensitivity was observed in the immediate (up to an hour) and 24 hours after the in-office bleaching. The popular desensitizing agent that could manage post-operative tooth sensitivity (TS) is 5% potassium nitrate and 2% sodium fluoride used before the in-office bleaching procedure.

## Introduction and background

A person's smile is one of the most striking features of their appearance; hence people want their smiles to look perfect. The hue of the teeth has a significant cosmetic value when it comes to smiling. Dark, brownish discolorations can be caused by pigments from beverages such as coffee, tea, tar from smoking, or constant betel nut chewing. In addition, excessive consumption of oranges, carrots, or chocolates may lead to food-related stains [1]. External bleaching, such as in-office bleaching, at-home bleaching, or a combination of both, can be done in such cases.

External tooth bleaching is a safe procedure that allows tooth whitening to be retained for several years. In-office bleaching or chair-side techniques are entirely dependent on the dental professional. Almost all the techniques involve applying a hydrogen peroxide gel of concentrations ranging from 25% to 40% [2]. Patients should undergo scaling and polishing of the tooth surface before bleaching and a careful evaluation to determine the nature of the discoloration. Nevertheless, concerns have been raised with introducing external bleaching, mostly related to the caustic effects of free oxygen radicals that may cause tissue damage in oral soft and hard tissues [2].

The presence of hypersensitivity is an indication of inflammatory changes in the dental pulp. Histologically, only a few pulps have demonstrated symptoms of inflammation post-in-office bleaching after the usage of hydrogen peroxide gels [2]. Yet, tooth sensitivity after in-office bleaching has been reported in up to 50% of cases. Most of the time, the tooth sensitivity followed by in-office bleaching is transient and lasts for 2 to 3 days [3]. Even if it is transient, this may discourage patients from completing the treatment.

At present, the mechanism resulting in tooth sensitivity followed by bleaching is unclear. The literature explains this is a result of hydrodynamic theory [3]. According to the aforementioned theory, the fluid movement inside the dentinal tubules stimulates the receptors in the pulp dentin area, causing pain [3]. Markowitz observed that the mechanism of tooth sensitivity due to dental bleaching, which occurs in healthy teeth with no other provocative stimulus, contradicts the explanation of the hydrodynamic theory, in which tooth sensitivity is caused when cold or tactile stimuli stimulate the exposed dentin [2]. Croll suggests that bleaching sensitivity results from fluid dynamics, where oxygen bubbles appear in the dentine tubules during the hydrogen peroxide application. This gas produced causes the dentinal fluid movements that lead to the activation of the intradental nerves [4].

According to various studies, it has been shown that bleaching agents can cause alterations on the dental surface, probably due to the low pH and the presence of chelating agents [5]. Bleaching agents can cause certain surface alterations on the enamel, such as depressions, surface porosity, and surface irregularities; this makes the dentin more susceptible to post-operative tooth sensitivity (PoS). In addition, the presence of flawed or leaky restorations, gingival recession, or defects in the cementoenamel junction can also cause severe tooth sensitivity post tooth bleaching [5,6]. Hence, the current study aimed to perform a systematic review to determine the efficacy of various desensitizing agents (DA) in managing post-operative tooth sensitivity and color alteration when applied before in-office bleaching procedures.

## Review

Focused question

Do desensitizing agents in randomized clinical trials help manage post-operative tooth sensitivity and color alteration when used before the in-office bleaching procedures?

Search strategy

The literature search was carried out for appropriate studies such as PubMed-MEDLINE, Scopus, and the Cochrane Central Register of Controlled Trials. Furthermore, sources like Google Scholar, and IndMed, along with the journal of web databases, were searched from the year 2009 up to April 30, 2021, lacking any limitations on language. In addition, ongoing clinical trials, including The National Institutes of Health Trials and Clinical Trial Registry India, were explored. Finally, the authors were contacted via mail for any grey literature. The search terms used by the authors were: “tooth bleaching agents,” “dentin desensitizing agents,” and “pain measurement/VAS.”

Eligibility criteria

(A) Randomized controlled clinical trials (RCTs) of vital in-office bleaching in a single or multi visits treatment using desensitizing agents. (B) Adult patients indicated for tooth whitening to improve the tooth shade were included. (C) Patients with fluorosis, tetracycline stains, caries, and restored or endodontically treated teeth were excluded. (D) A visual analogue scale (VAS) was used for pain measurement.

Screening and selection

The articles were screened by title and consequently by abstract by two reviewers (KK & AST) independently. The search did not include narrative or historical reviews, letters, or case reports. Full-text reading was conducted only on those articles that presented the keywords either in the title or abstract, or both. To screen the full text for the eligibility of the papers sans abstract but titles evincing of objectives which were related to the eligibility criteria were selected. After the primary selection of papers, the two reviewers thoroughly read the full-text articles (KK & AST). Post this, the articles that fulfilled all the eligibility criteria of this review were processed for further data extraction. Finally, both the reviewers (KK & AST) went through all the selected article’s references to search for additional pertinent articles. All disagreements were resolved through a discussion amongst the reviewers. Yet, any persisting disagreements were judged by the third reviewer and were considered conclusive. 

Quality assessment

Two reviewers (KK & AST) analyzed the risk of bias in every trial using the Cochrane Collaboration’s tool for assessing the risk of bias [7], with the consensus resolving any arguments.

Data extraction

Two authors (KK & AST) made use of the Microsoft Excel software (Microsoft, Redmond, WA, USA) and specially designed data extraction forms to independently extract data. The authors maintained discussion among them to help resolve any disagreement. For each selected study, the following data were then excerpted from a standard form (when available). These included author and year of publication, study design, sample size, participant’s characteristics, desensitizing agent, comparator, hydrogen peroxide (%), attrition, analgesics, attrition, and author’s conclusion.

Outcomes

Post-operative sensitivity experienced by the participants during and after the in-office bleaching procedure was the primary outcome. We considered the postoperative tooth sensitivity after 1 hour and 24 hours. The varying responses regarding different desensitizing agents were recorded.

The secondary outcome of this review was to assess the difference in tooth color when a desensitizing agent was used before the bleaching procedure. We considered clinical trials measuring the color change in delta shade guide units or color change in delta E (CIEL*a*b*) after bleaching.

Results

**Figure 1:**
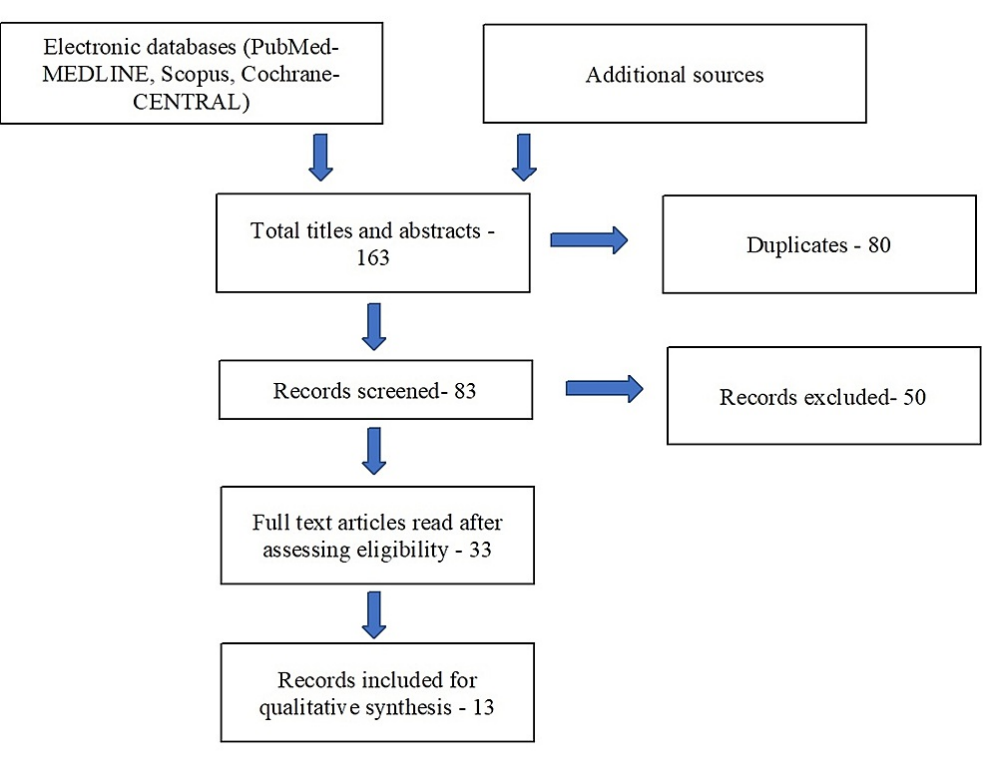
Flowchart summarizing the article selection process

The reporting is based on the Preferred Reporting Items for Systematic Reviews and Meta-Analyses (PRISMA) guidelines [8]. (Figure 1)

Prisma guideline

The terms “tooth bleaching agents,” “dentin desensitizing agents,” and “pain measurement/VAS” were searched for in various databases. The PubMed-Medline, Scopus, Cochrane Library, and additional sources identified 163 search results, out of which 80 were duplicates. The remaining 83 unique studies were screened for the titles and abstracts, and 33 articles were selected for full-text screening. Thirteen articles [9-21] that matched the eligibility were filtered for data extraction (n = 13). An outline of the selected papers and their conclusions is presented in Table 1. the exclusion criteria are also mentioned in Table 2.

**Table 1:** Overview of the studies processed for data extraction RCT: randomized controlled trial, CPP-ACP: Casein phosphopeptide–amorphous calcium phosphate complexes, DCPA: dicalcium phosphate anhydrous, TTCP: tetra calcium phosphate, DA: Desensitising agent, PoS: post-operative sensitivity

Author & Year of Publication	Study design	Sample size in each group	Hydrogen peroxide (%)	Attrition	Desensitising agent (DA)	Placebo group	Author’s Conclusion
Tay LY et al. 2009 [9]	Double blind RCT, Parallel study design	15	35	0	5% potassium nitrate & 2% sodium fluoride	Same composition as the Dag without the active components	The use of concerned DA before tooth bleaching procedure using 35% concentration of hydrogen peroxide can reduce tooth sensitivity without jeopardizing the efficacy of in-office bleaching.
A Reis et al. 2011 [10]	Double blind RCT, parallel study design	-	35	0	5% potassium nitrate & 2% sodium fluoride	Same composition as the DA without the active components	The use of the concerned DA before a light-activated tooth bleaching procedure using a 35% concentration of hydrogen peroxide can reduce tooth sensitivity without jeopardizing the efficacy of in-office bleaching.
M Pale et al. 2013 [11]	Parallel study design	16	28	0	5% potassium nitrate and glycerin	Glycerin in a custom-made tray	The use of the concerned DA before in-office bleaching, using 28% concentrated hydrogen peroxide, the PoS was reduced significantly, but the results of the bleaching efficacy were also decreased.
CH Thiesen et al. 2015 [12]	Double blinded RCT, Parallel study design	15	35	0	Colgate Sensitive Pro-Relief, Sensory experience ProEnamel	Colgate Total	The use of dentifrice containing DA reduced PoS and did not affect the effectiveness of in-office bleaching by 35% concentrated hydrogen peroxide when compared with the control group.
RK Henry et al. 2015 [13]	Double blind RCT, Parallel study design	10	30	2	Sugar-free gum with 0.6% CPP-ACP	No gum, Sugar-free gum without CPP, ACP	Chewing gum with DA before the bleaching procedure using 30% hydrogen peroxide did not show any significant change in PoS.
AD Loguercio et al. 2015 [14]	Randomized, parallel, double blinded, placebo-controlled trial	20	35	0	Nano-P paste	Carbopol-based gel mixed in water and thickener	The use of the concerned DA used before in-office bleaching with 35% concentrated hydrogen peroxide did not reduce PoS.
D Mehta et al. 2017 [15]	Randomized, placebo-controlled, single masked, spilt mouth study	25	40	2	DCPA and TTCP containing paste. DCPA: dicalcium phosphate anhydrous TTCP: tetra calcium phosphate	Analogous placebo paste	Application of the concerned DA before bleaching using 40% hydrogen peroxide on the enamel surface largely prevented PoS.
Diniz et al. 2018 [16]	Randomized, single blind, split-mouth clinical trial	33	35	0	Gluma desensitiser liquid	Same composition as the DA except for the absence of the active ingredients	Prior application of the concerned DA to tooth bleaching procedure using 35% hydrogen peroxide did not significantly reduce PoS and did not jeopardize the whitening effect of the bleaching procedure.
Oldonini et al.2018 [17]	Parallel study design	40	30	0	10% ACP	None	The use of the concerned DA reduced PoS after in-office treatment using 30% hydrogen peroxide.
Parreiras et al. 2018 [18]	Triple blind RCT, Parallel study design	42	35	1	5% potassium nitrate, 5% glutaraldehyde in a hydroxy cellulose gel	Same composition as the DA except for the absence of the active ingredients	The DA was effective in reducing the PoS of bleaching-induced tooth sensitivity (using 35% hydrogen peroxide) without jeopardizing the whitening results.
Paula et al. 2018 [19]	Randomized, controlled, double-blind & split mouth study	48	35	0	5% potassium nitrate gel	Same composition as the DA except for the absence of the active ingredients	The desensitising agent composed of 5% potassium nitrate gel reduced PoS, after bleaching with 35% hydrogen peroxide.
Martini et al. 2020 [20]	Triple blind RCT, Parallel study design	90	35	1	5% potassium nitrate and 2% sodium fluoride	Same composition as the DA except for the absence of the active ingredients	5% potassium nitrate was not effective in reducing PoS using 35% hydrogen peroxide.
Rezende et al. 2020 [21]	Triple blind RCT, Split mouth study	43	35	0	10% potassium nitrate	Same composition as the DA except for the absence of the active ingredients	Application of the concerned DA before in-office bleaching did not reduce PoS and did not jeopardize color change.

**Table 2:** Criteria for exclusion

Reasons for Exclusion
14 studies: failed to meet the eligibility criteria.
4 studies: irrelevant information
2 studies: missing information

Study design and subject characteristics

Using the Cochrane Collaboration’s tool for assessing the risk of bias with the consensus resolving any arguments, two reviewers (KK & AST) analyzed the risk of bias in every trial.

All selected studies were randomized clinical trials. Eleven studies [9-15,17,18,20] were performed using a parallel study design, whereas the other three [16,19,20] were performed as a split-mouth study. In addition, two studies [15,16] were single masked, three studies [18,20,21] were triple masked, whereas six studies [9,10,12-14,19] were double-masked. In all the selected studies, the study population was patients with caries and restoration-free teeth indicated for the tooth bleaching procedure. All thirteen studies were conducted in the adult population in a clinical setting.

The review included 841 adult patients with vital pulp status who received an intervention of a DA before the in-office bleaching procedure. The in-office bleaching procedure was done in multiple sessions. The in-office bleaching procedure and concentration varied between the included studies. One study [11] used 28% of hydrogen peroxide (H2O2) concentration of bleaching agent, two studies [13,17] used 30% concentration, nine studies [9,10,12,14,16,18,20,21] used 35% and two studies [15] used 40% H2O2 concentration of bleaching agent.

In the study by RK Henry et al., sugar-free chewing gum containing a desensitizing agent was given to the participants to chew for seven days before the in-office bleaching procedure [13]. CH Thiesen et al. used desensitizing dentifrice 15 days before the in-office bleaching procedure [12]. The remaining studies applied a desensitizing agent over the tooth surface in accordance with their respective manufacturer’s instructions, followed by an in-office bleaching procedure [9,10,14-21]. Analgesics were used to manage postoperative pain in one study within 24 hours [21].

The post-operative pain was evaluated as post-operative tooth sensitivity as risk and post-operative tooth sensitivity as intensity. The former evaluated the incidence of experiencing post-operative tooth sensitivity, whereas the latter evaluated the intensity of the tooth's sensitivity. The results extracted as tooth sensitivity experienced by the participants as the risk were included in the systematic review.

Meta-analysis was not possible due to varied outcome units and heterogenous studies. Forest plots are not added since a meta-analysis was not done. A pooled estimate cannot be calculated with different outcome units and heterogenous studies.

Pain scores immediately after in-office bleaching

Pain scores immediately after the in-office bleaching procedure were investigated in 11 studies [7-10,13,15,16]. The participants receiving prior desensitizing agent applications reported significantly lower pain scores in the VAS reports. Since the concentration of H2O2 can influence post-operative sensitivity, the p-values were evaluated in different categories according to the concentrations of the bleaching agent used, as provided in Table 1. According to the desensitizing agents used, there was a statistical difference between potassium nitrate (KNO3) and 2% sodium fluoride (NaF) desensitizing gel (Desensibilize KF 2%) and the rest of the desensitizing agents reported in Table 1. Therefore, the 5% KNO3 and 2% NaF desensitizing gel is the most often considered intervention. However, there was no significant difference between the use of 10% amorphous calcium phosphate (ACP) and Gluma Desensitizer Liquid (Heraeus Kulzer GmbH) SensodyneÒ ProNamelä (GSK, USA) [16]. The use of desensitizing dentifrice (Sensodyne ProNamel) for 15 days before the in-office bleaching procedure had shown a significant difference in the post-operative tooth sensitivity experienced by the participants [12].

Pain scores at 24 hours

Pain scores at 24 hours after the in-office bleaching procedure were investigated in nine studies [10-12,15,16,18-21]. Patients receiving the application of desensitizing agents reported significant differences in a few studies [10,11,15,18]. Six studies showed no significant differences between the placebo and the desensitizing agents [13,16,19-21]. The incidence of post-operative sensitivity was lesser than the tooth sensitivity (TS) experienced immediately after the bleaching procedure in both the placebo and intervention groups [10,11,15,18].

Discussion

The present systematic review included 13 RCT’s examining the effectiveness of various desensitizing agents and placebo with preceding application to the in-office bleaching procedure [9-21]. This resulted in a remarkably notable difference in the post-operative tooth sensitivity experienced immediately after the bleaching procedure [9,10,11,12,15,17,18]. The studies included in the current synthesis differed regarding the desensitizing agents used, the mode of application of the desensitizing agent, and the concentration of the bleaching gel used. For simplicity and clarity, the outcomes were categorized according to the concentration of the bleaching agents. After the systematic analysis of all the articles included in this review, the most used concentration of bleaching agent was considered. This was composed of a 35% H2O2 concentration of bleaching agent, and the direct gel application on the tooth surface being as it is the most efficacious mode of application of the desensitizing agent. This mode of application of desensitizing agent was considered in particular with the reason being that desensitizing chewing gums and desensitizing dentifrice have fewer studies to claim validity and potency by comparing their mode of application [12,13].

Immediately after the in-office bleaching procedure, a significant reduction in the incidence of post-operative sensitivity was experienced with the use of 5% KNO3 with 2% NaF (Desensibile KF 2%) [9,10,11,19-21], 10% ACP [13,14], and 5% potassium nitrate with 5% glutaraldehyde in a hydroxyethylcellulose gel [18]. A significant reduction in the post-operative tooth sensitivity was observed in the first 24 hours of the same [10,11,15,18]; however, not much difference between the placebo and the desensitizing agents was observed following 48 hours [16,19-21].

Post-operative tooth sensitivity is a common problem experienced by patients after vital bleaching. Since the tooth bleaching procedure usually requires multiple sessions to achieve the required shade, sore tooth sensitivity experienced by the patients during the first visit could be a factor for patients to be discouraged from continuing the treatment [22-26]. Several studies have called attention to the role of desensitizing agents’ application in the management of post-operative tooth sensitivity in vital in-office bleaching procedures [22-31]. Since the concentration of the bleaching agent is considerably higher than in at-home bleaching procedures, the experience of post-operative tooth sensitivity is also higher [31], with a lack in more comparison studies. From the data available so far, it could be understood that the most effective mode of application of the desensitizing agent would be the application of the agents directly on the tooth surface before the in-office bleaching procedure [25,26].

Tooth sensitivity should be distinguished from PoS experienced after the tooth bleaching procedure. Investigators have theorized that there could be some degree of pulpal inflammation associated with the vital bleaching procedure; otherwise, the PoS experience is thought to be related to the hydrodynamic theory [25,26,30,31]. Vital bleaching, an especially higher concentration of bleaching agent, might release cell-derived factors such as prostaglandins and ATP. This interaction could excite the pulpal nociceptors and eventually lead to pulpal tissue damage [25,26,30,31].

In a study by A Reis et al., the use of 5% KNO3 with 2% NaF did not produce any significant change immediately after the in-office bleaching procedure. Still, it was observed that during the first 24 hours, there was a remarkable reduction in the post-operative PoS intensity experienced in the experimental group in comparison to the placebo group [8]. During the in-office bleaching procedure, the higher the bleaching agent's concentration, the greater the enamel and dentin permeability, which can cause higher PoS experienced by the patient than at-home vital bleaching techniques [4,15,25,29,20,31]. The exact mechanism of KNO3 and NaF action as a DA is not well understood. The potassium ions that are most likely to be the active component works by lowering the sensitivity activity of the dentine by the depolarization action of potassium. Whereas fluorides occlude the dentinal tubules, which are exposed or reduce the flow of dental lymph towards the pulp, effectively halting the transmission of stimuli [32].

The clinical significance of applying the desensitizing agent before the in-office bleaching procedure is to prepare the tooth surface to be resistant to TS in an action of the higher concentration of the bleaching agents, especially in cases of in-office bleaching procedure. A higher concentration of bleaching agent is applied for short periods, mostly three 15 minutes sessions for a total of 45 minutes. This increases a higher risk of the incidence of post-operative TS experienced by the patient [25,26,30,31]. Application of desensitizing agent before the in-office bleaching could manage areas of thin enamel such as pits, attrition, abrasion, craze lines, and other developmental anomalies where the dentinal tubules are closer to the outer enamel surface or are possibly exposed [5,6]. Several studies used different desensitizing agents such as 10% ACP [13,14], 5% potassium nitrate and 2% sodium fluoride [19,20,21], and 5% potassium nitrate with 5% glutaraldehyde in a hydroxyethylcellulose gel [18] had shown a significant reduction in post-operative TS with its prior use to the in-office bleaching procedure.

Regarding the color change, efficacy alteration due to the prior application of desensitizing agents was found to be void in the 14 studies assessed in this systematic review [9-16,18-21]. The limitation of this systematic review is a lack of standardization of evaluation and study conduction procedures amongst the various research studies selected. In many studies, the placebo and the intervention group were conducted on different individuals. In post-operative sensitivity, a split-mouth study design would have procured more accurate readings of the interventions applied. In a few studies, regardless of the intervention, a bleaching agent with a desensitizer was used. This could hinder the true value of the interventional desensitizer used.

## Conclusions

Bleaching agents can cause certain surface alterations on the enamel, such as depressions, surface porosity, and surface irregularities; this makes the dentin more susceptible to post-operative tooth sensitivity (PoS). In addition, the presence of flawed or leaky restorations, gingival recession, or defects in the cementoenamel junction can also cause severe tooth sensitivity post tooth bleaching. Hence, the current study aimed to perform a systematic review to determine the efficacy of various desensitizing agents (DA) in managing post-operative tooth sensitivity and color alteration when applied before in-office bleaching procedures. The most significant reduction of post-operative sensitivity was observed in the immediate (up to one hour) and 24 hours after the in-office bleaching. Therefore, more studies are required for the prior use of dentifrices to manage post-operative sensitivity after in-office bleaching. The popular desensitizing agent that could manage post-operative TS is 5% potassium nitrate and 2% sodium fluoride used before the in-office bleaching procedure. However, newer desensitizing agents must be researched with improved randomized clinical trials for a better assessment of their efficacy. 
